# Spinal Inhibition of GABAB Receptors by the Extracellular Matrix Protein Fibulin-2 in Neuropathic Rats

**DOI:** 10.3389/fncel.2020.00214

**Published:** 2020-07-15

**Authors:** Marie-Amélie Papon, Yves Le Feuvre, Gabriel Barreda-Gómez, Alexandre Favereaux, Fanny Farrugia, Rabia Bouali-Benazzouz, Frédéric Nagy, Rafael Rodríguez-Puertas, Marc Landry

**Affiliations:** ^1^Institut Interdisciplinaire de Neurosciences, University of Bordeaux, Bordeaux, France; ^2^CNRS UMR 5297, Institut Interdisciplinaire de Neurosciences, Bordeaux, France; ^3^Department of Pharmacology, University of the Basque Country UPV/EHU, Leioa, Spain

**Keywords:** fibulin-2, disinhibition, GABAB receptor, neuropathic pain, spinal cord

## Abstract

In the central nervous system, the inhibitory GABAB receptor is the archetype of heterodimeric G protein-coupled receptors (GPCRs). Receptor interaction with partner proteins has emerged as a novel mechanism to alter GPCR signaling in pathophysiological conditions. We propose here that GABAB activity is inhibited through the specific binding of fibulin-2, an extracellular matrix protein, to the B1a subunit in a rat model of neuropathic pain. We demonstrate that fibulin-2 hampers GABAB activation, presumably through decreasing agonist-induced conformational changes. Fibulin-2 regulates the GABAB-mediated presynaptic inhibition of neurotransmitter release and weakens the GABAB-mediated inhibitory effect in neuronal cell culture. In the dorsal spinal cord of neuropathic rats, fibulin-2 is overexpressed and colocalized with B1a. Fibulin-2 may thus interact with presynaptic GABAB receptors, including those on nociceptive afferents. By applying anti-fibulin-2 siRNA *in vivo*, we enhanced the antinociceptive effect of intrathecal baclofen in neuropathic rats, thus demonstrating that fibulin-2 limits the action of GABAB agonists *in vivo*. Taken together, our data provide an example of an endogenous regulation of GABAB receptor by extracellular matrix proteins and demonstrate its functional impact on pathophysiological processes of pain sensitization.

## Introduction

G protein-coupled receptors (GPCRs) modulate a wide range of physiological processes and new drug candidates are continually being developed for selective GPCRs (Lagerstrom and Schioth, 2008; Hauser et al., 2017; Topiol, 2018). Functional GABAB receptors are expressed as obligate heterodimers of the two subunits GABAB1 (B1) and GABAB2 (B2) (Jones et al., 1998; Kaupmann et al., 1998), and GABAB-mediated inhibition is an essential control of neuronal activity throughout the central nervous system. GABAB receptor subunits bind several partner proteins (Bettler et al., 2004; Pin and Bettler, 2016; Fritzius et al., 2017) that regulate the receptor activation in pathophysiological conditions such as chronic pain (Laffray et al., 2007; Laffray et al., 2012; Zemoura et al., 2016; Malcangio, 2018). Among these binding partners, extracellular matrix (ECM) proteins have been shown to regulate postsynaptic GABAB-mediated inhibition (Saghatelyan et al., 2003). However, the importance of such interaction in a pathophysiological context has not been established yet.

In the central nervous system, aggregates of ECM molecules appear as so-called perineuronal nets that surround soma and dendrites (Fawcett et al., 2019). These nets are heterogeneous in structure and composition, incorporating proteins derived from both neuronal and glial cells. The expression of ECM proteins is developmentally and spatially regulated in the central nervous system. Some of these molecules are up-regulated following adult nerve injury (Quraishe et al., 2018; Roumazeilles et al., 2018) and ECM proteins have been implicated in synaptic differentiation and plasticity (Rowlands et al., 2018). The emerging mechanisms comprise clustering of receptors and changes in their composition and function (Dityatev and Schachner, 2006; Ferrer-Ferrer and Dityatev, 2018). To date, beside the specific class of adhesion GPCR (Langenhan et al., 2016) only very rare studies have considered interactions between ECM proteins and GPCRs (Saghatelyan et al., 2003; Yeh et al., 2008) and the mechanisms by which ECM proteins regulate GPCR activation in neurons remains largely unknown.

In this context, the present study focuses on the role of fibulin-2, an extracellular binding partner of the B1a variant of GABAB1 subunit (Blein et al., 2004) that is mainly found in presynaptic compartments (Vigot et al., 2006). We demonstrate that fibulin-2 up-regulation hampers GABAB activation and signaling by agonists. In contrast, we show that the loss of fibulin-2 facilitates agonist-induced activation of the GABAB receptor and decreases the efficiency of antagonists. Therefore, we suggest that fibulin-2 may limit conformational changes that are necessary for the receptor activation, and facilitate an inactive conformation of the receptor.

As a functional consequence of this mechanism, we demonstrate in a primary cell culture model that fibulin-2 regulates the GABAB-mediated presynaptic inhibition of neurotransmitter release. We further show the physiological impact of this mechanism on neuropathic pain sensitization in the dorsal horn of the rat spinal cord. Our data indicated fibulin-2 up-regulation in the dorsal spinal cord of neuropathic rats. In such chronic pain conditions, the efficiency of GABAB-mediated inhibition was improved by knocking-down the protein expression with *in vivo* application of anti-fibulin-2 small interference (si) RNA.

Finally, our demonstration that the fibulin-2 endogenous extracellular matrix protein inhibits GPCR activity in a pain context highlights a novel mechanism of disinhibition, and provides the groundwork for further studies of pathophysiological regulations.

## Materials and Methods

### Primary Cell Cultures

Neuronal cells were obtained from rats (Sprague Dawley) at 18 days of embryonic development. The embryos were delivered by cesarean section from deeply anesthetized rats and killed by decapitation. Pieces of lumbar region of the spinal cord or cortex were exposed to a 1X trypsin solution (Gibco) for 25 min at 37°C. These pieces were then mechanically dissociated by forcing them through fine-tipped pipettes several times. Cells were plated on glass coverslips at a density of 150,000/100 μL. The glass coverslips were previously coated overnight at 37°C with a 0.1 mg/mL polylysine solution for cortex and 1 mg/mL for spinal cord (Sigma, Saint Louis, MO, United States) and for 2 h with a 0.05 mg/mL laminine solution (Sigma). The cultures were kept in Serum-free Neurobasal TM medium (Gibco), supplemented with B27 (Gibco) and Glutamax (Gibco). Half of the medium was changed twice a week.

Dissociated rat spinal neurons, cultured in Serum-free Neurobasal TM medium (Gibco, ThermoFisher, Waltham, MA, United States), were transfected with 400 ng plasmid DNA/2 μL Lipofectamine 2000 (Invitrogen, Cergy Pontoise, France). In most cases, transfected cells incorporated all cDNAs together. siRNA was added 24 h after transfection with cDNAs (500 ng siRNA/3μL of Lipofectamine 2000). Growth medium was replaced after 6 h and cells were harvested 36 h after siRNA transfection.

### Plasmids and siRNAs

For Fluorescence (or Förster) Resonance Energy Transfer (FRET) experiments, B1a/B1b, and B2 cDNA were modified at their N-terminus by the addition of Green Fluorescent Protein (GFP), or tandem-dimer discosoma red fluorescent protein (t-dimer DsRed), respectively (Laffray et al., 2007, 2012). DsRed-GABAB2 was subcloned in the pcDNA3.1 mammalian expression vector. GABAB1a/b were subcloned in the pEGFP-C3 to express GFP at the N-terminus. For co-immunoprecipitation experiments, myc-GABAB1a/b and HA-GABAB2 expressing plasmids were transfected with Lipofectamine 2000 (Invitrogen) in COS-7 cells as described previously (Laffray et al., 2012). The Rc/CMV vector containing the full-length fibulin-2 construct was obtained from Dr. Takako Sasaki (Max-Planck-Institut, Martinsried, Germany) and was prepared as described (Pan et al., 1993; Sasaki et al., 1995, 1996). Fibulin-2-Flag was co-expressed in COS-7 cells with hemagglutinin (HA)-GABAB2 and myc-GABAB1a/b. Anti-fibulin-2 siRNA was a pool of 3 target-specific 20-25 nucleotides siRNAs (sc-43120, Santa Cruz Biotechnology, Santa Cruz, CA, United States). Mismatch siRNA was a non-targeting 20–25 nucleotides designed as a negative control (sc-37007, Santa Cruz Biotechnology).

The efficacy of siRNA to downregulate the expression of fibulin-2 mRNA was assessed by SYBRGreen-based quantitative reverse transcription – polymerase chain reaction (qRT-PCR) both on cell cultures (data not shown) and after intrathecal injections *in-vivo* (see Results section, Expression of Fibulin-2 *in vivo*).

### qRT-PCR

Expression analysis of fibulin-1 and fibulin-2 mRNA was performed with the DyNAmoTM SYBR Green qPCR kit (Finnzymes, Espoo, Finland). Triplicate runs were performed and Succinate dehydrogenase Complex, Subunit A (SDHA) was used as a normalizer. The relative level of expression was calculated using the comparative (2ΔΔCT) method (Livak and Schmittgen, 2001).

### Spectral and Lifetime Correlated Acquisitions

Spinal cultures were treated for 1 h or 15 min at 37°C with fibulin-2 protein at 25 or 50 nM. Then, cultures were treated for 30 min at 37°C with baclofen (10^–3^ M to 10^–9^ M, Sigma B5399), or with GABA (10^–4^ M, Sigma A5835); or with competitive antagonists CGP55485 (0.5 or 50 μM, Tocris 55845) or saclofen (100 μM or 1 mM, Sigma S166) co-applied with baclofen. Next, cells were fixed 20 min at 37°C [4% paraformaldehyde, 4% sacharose in phosphate buffer (PB) 0.1M, pH 7.4], washed [0.1M PB saline (PBS), 10 min at RT] and mounted on slides (Fluorescent Mounting Medium Dako Cytomation, Agilent, Santa Clara, CA, United States).

#### Two-Photon-Fluorescence Lifetime Imaging Measurement (2P-FLIM)

The interaction between GFP-tagged proteins and t-dimer DsRed-tagged proteins was studied as described previously (Laffray et al., 2007, 2012) using quantitative FRET (Fluorescence or Förster Resonance Energy Transfer) determination with FLIM (Fluorescence Lifetime Imaging Measurements) using Time Correlated-Single Photon Counting (Becker and Hickl, Berlin, Germany). To assess protein interactions, we calculated a relative FRET efficiency for each acquisition as follow: FRET Efficiency (%) = (τ_Dmean_-τ_DA_)/τ_Dmean_ × 100 where τ is the time constant of the exponential fit with τDmean being the mean lifetime of the donor fluorophore (GFP) when expressed alone and τDA being the lifetime of the donor fluorophore in the presence of the acceptor (t-dimer DsRed). Results were expressed as a mean FRET efficiency (*n* = 21 cells or more in each condition) ± SEM.

Due to the mono-exponential decay of GFP and their spectra compatibility, the GFP-DsRed couple is very well suited for quantitative FRET determination with FLIM (Fluorescence Lifetime Imaging Measurements) (Tramier et al., 2002; Stubbs et al., 2005). Moreover, the t-dimer DsRed is a tandem of monomeric red fluorescent protein that matures rapidly, does not form aggregates, and has minimal emission when excited at 900 nm, a wavelength optimal for GFP and minimizing the excitation of t-dimer DsRed (Campbell et al., 2002).

The interaction between GFP-tagged protein and t-dimer DsRed-tagged protein was studied using two-photon-Fluorescence Lifetime Imaging Measurement (2P-FLIM) with the SPCM and SPC Image software (Becker & Hickl).

For GFP excitation, we used a two-photon pulsed excitation (890 nm), given by the Coherent Mira 900-F laser 5W (Coherent inc. Laser Group, Santa Clara, CA, United States). All acquisitions were undertaken with a 100x, 1.4NA, Leica oil objective lens on a Leica TCS SP2 inverted confocal microscope. The laser repetition frequency was 76 MHz which gave a 12 ns temporal window for lifetime measurements. Photon detection was performed in cells using the SPRC160, a homemade system based on a 16 channel multi-anode photomultiplier head (PML16, Becker&Hickl) and a concave holographic grating allowing spectral dispersion on the detector. The Becker & Hickl TCSPC 730 card (Becker & Hickl, Berlin, Germany) determined the time between fluorophore excitation and photon emission, as well as routing information associated to spectral range. This card was driven by the SPCM software which allowed fluorescence decay curve measurements using a single-spot mode; and fluorescence decay curve fits, allowing lifetime determination were obtained using the SPC Image software (both software from Becker & Hickl). The choice between mono-exponential decay and bi-exponential decay fitting was done using the reduced χ2 parameter. Lifetime values were extracted from three spectral channels, from 494 to 542 nm, and averaged. To ensure reliability of our measurements, we checked the lifetime stability upon this spectral range. As a second control, the first channels were used to ensure no autofluorescence in measured cells, which otherwise could result in artefactual FRET positive response.

### Co-immunoprecipitation

The co-immunoprecipitation experiments were performed in COS-7 cells after transfection, according to the previously described procedure (Laffray et al., 2012). Briefly, total protein extract was homogenized in ice-cold PBS containing 1% CHAPS, 25 mM Hepes and 150 mM NaCl, and protease inhibitors, pH 7.4. Homogenates were incubated overnight with 50 μl of magnetic beads (protein-A sepharose, Sigma) at 4°C. After centrifugation, the supernatants were transferred to the tube containing 5 μg of specific anti-myc (Roche) or anti-flag (Sigma) antibodies. Then, samples were washed twice, incubated in Laemmli buffer (Sigma) and subjected to Western blot detection. After electrophoresis using sodium dodecyl sulfate – polyacrylamide gel electrophoresis (SDS–PAGE), samples were transferred to polyvinylidene difluoride (PVDF) membranes. Blots were blocked in 5% skimmed milk for 30 min and then incubated with antibodies (mouse anti-myc, 1/1000, Roche; guinea pig anti-flag, Sigma; mouse anti-GABAB2, 1/1000, Eurobio, Les Ulis, France) at 4°C overnight. Then detection was performed with the appropriate HRP-conjugated secondary antibodies at 1/1000 for 1 h30 (anti-mouse or anti-guinea-pig, Dako products, Agilent). Immunoreactivity was developed using the enhanced chemiluminescent method and visualized with a Syngene device (ChemiGenius 2XE model; Synoptics Ltd, Cambridge, United Kingdom).

### Immunohistochemistry on Tissue Sections

Rats were perfused with 4% paraformaldehyde. For electron microscopy, glutaraldehyde (0.2%) was added to the fixative solution. The lumbar spinal cord was rapidly dissected out, and post-fixed for 2 h in 4% paraformaldehyde. Tissue samples were then processed for light or electron microscopy.

For light microscopy, transverse spinal cord sections were cut at 30 μm thickness on a cryostat (Microm) and processed for double-fluorescent labeling experiments. Sections were incubated overnight at 4°C with a goat anti-fibulin-2 (1/400, Santa Cruz) and a sheep anti-GABAB1a (1/20,000; gift from Dr. Andrew Calver, GlaxoSmith Kline, Harlow, United Kingdom). Subsequently, GABAB1a was visualized with a biotinylated secondary antibody and a tyramide-based method (Landry et al., 2004) using FITC-conjugated tyramide (PerkinElmer Life Sciences, Boston, MA, United States). Fibulin-2 was detected with a biotinylated secondary antibody and Alexa568-conjugated streptavidin (Molecular Probes Inc., Eugene, OR, United States).

For co-detection of fibulin-2 and calcitonin gene-related peptide (CGRP), sections were incubated overnight at 4°C with goat anti-fibulin-2 (1/400, Santa Cruz) and rabbit anti-CGRP (1/1000, Peninsula). Subsequently, fibulin-2 was detected with biotinylated secondary antibody and Alexa568-conjugated streptavidin (Molecular Probes Inc., Eugene, OR, United States). CGRP was visualized with Alexa 488-conjugated anti-rabbit (Molecular Probes Inc., Eugene, OR, United States). Sections were viewed with a confocal microscope (Leica DMR TCS SP2 AOBS, Mannheim, Germany).

For electron microscopy, Vibratome^®^ sections were cut at 200 μm thickness and samples of superficial spinal cord laminae (I-II) were embedded in Lowicryl^®^ HM20 resin (EMS, Hatfield, PA, United States) with the progressive lowering of temperature method, using the Reichert AFS system (Leica, Wien, Austria) according to the instruction manual. Ultrathin sections (70 nm) from Lowicryl blocks were collected on butvar-coated single-slotted nickel grids (EMS) and submitted to postembedding immunogold multiple labeling. GABAB receptor subunits and fibulin-2 protein were detected with: anti-GABAB1a (1/100, sheep, Glaxo), and anti-fibulin-2 (1/500, goat, Santa Cruz) primary antibodies and visualized with the appropriate colloidal gold-conjugated detection systems (BBI, Cardiff, United Kingdom; EMS) (GABAB1a = 15 nm, fibulin-2 = 5 nm). Grids were rapidly fixed for 1 min in 1% glutaraldehyde diluted in water. After rinsing, grids were finally stained with 2% uranyl acetate in water for 12 min and examined with a Hitachi H7650 electron microscope (Elexxience, Verrières-le-buisson, France) equipped with an Orius SC1000 camera (GATAN, Evry, France).

CGRP and fibulin-2 protein were detected with anti-CGRP (1/500, rabbit, Peninsula), and anti-fibulin-2 (1/500, goat, Santa Cruz) primary antibodies and visualized with the appropriate colloidal gold-conjugated detection systems (BBI, Cardiff, United Kingdom; EMS) (CGRP = 5 nm and fibulin-2 = 15 nm).

Immunostaining of the spinal cord was visualized under a SPE confocal microscope (Leica Microsystems). Images to be compared were collected during the same session using identical scanning settings. They were then imported into “ImageJ” free software (version 1.42q) (NIH, Bethesda, MA, United States) for quantitative analysis. Background was subtracted by thresholding. For fibulin-2 and GABAB1a intensity assessment, the mean gray level corresponding to fluorescence intensity was measured (Zaky et al., 2018). Results were expressed as a percentage of the intensity in sham animals. Counting was performed on 5 sections per rat, 8 (fibulin-2) or 4 (GABAB1a) rats per condition (sham or SNL). The extent of colocalization between fibulin-2 and GABAB1a was quantitatively assessed using a semi-automatized procedure with the Image J software (Landry et al., 2004; Zaky et al., 2018). After thresholding, colocalization area was assessed in the entire fields acquired in the dorsal horn of the spinal cord (4 sections per rat, 8 rats per condition). Results were expressed as a percentage of GABAB1a total labeling that is seen in the area.

### Patch Clamp

For voltage-clamp recordings, 8–10 days-aged cultured spinal neurons on their coverslip were submerged in normal Krebs solution containing 130.5 mM NaCl, 2.4 mM KCl, 2.4 mM CaCl_2_, 19.5 mM NaHCO_3_, 1.3 mM MgSO_4_, 1.2 mM KH_2_PO_4_, 1.25 mM HEPES, 10.0 mM glucose, pH 7.4, equilibrated with 95% O_2_ and 5% CO_2_, at room temperature (20–23°C). The coverslip was placed in a chamber and visualized by means of infrared DIC video-microscopy using an upright microscope BX51WI (Olympus France, Rungis, France) equipped with a 40x water immersion lens (LUMPlanFI/IR), and a CCD camera C2400-03 (Hamamatsu, Hamamatsu City, Japan). The culture was continuously superfused with Kreb solution at 1.6 mL.mn^–1^. Patch pipettes (6–8 M Ω) were filled with 120 mM potassium gluconate, 20 mM KCl, 0.1 mM CaCl_2_, 1.3 mM MgCl_2_, 1 mM EGTA, 10 mM HEPES, 0.1 mM GTP, 0.2 mM cAMP, 0.1 mM leupeptin, 3 mM Na_2_-ATP and 77 mM D-mannitol, pH 7.3. Series resistance was negligible.

Miniature EPSCs were recorded in voltage clamp mode (holding potential −60 mV) in the presence of 10^–6^ M Tetrodotoxin (TTX). In such experimental conditions, no action potential could be evoked following injection of positive current into the recorded neuron, demonstrating a complete blockade of action potential propagation. Signals were amplified using an Axoclamp 2B amplifier and digitized at 10 kHz. Individual events were extracted from raw data using mini-analysis software^1^ over a duration of 2 min and visually filtered according to their general shape.

### GTPγS Binding and Competition Binding Assay

#### Membrane Preparation

Primary cell cultures of rat cortex (8 million of cells/mL) were homogenized using a Teflon glass grinder (10 up-and-down strokes at 1500 rpm) in 5 ml of homogenization buffer (1 mM EGTA, 3 mM MgCl_2_ and 50 mM Tris-HCl, pH 7.4) supplemented with 0.25 mM sucrose. The crude homogenate was centrifuged for 5 min at 500 rpm (4°C) and the supernatant was recentrifuged for 10 min at 18000 rpm (4°C). The resultant pellet was washed in 4 ml of homogenization buffer and recentrifuged in similar conditions. Aliquots of protein were stored at –80°C until assay. Protein content was measured according to the method of Bowery (2006) using bovine serum albumin (BSA) as standard.

#### [^35^S]GTPγS Binding Assays

Membrane aliquots were thawed and resuspended in a buffer containing 1mM EGTA, 3 mM MgCl_2_, 100 mM NaCl, 3 mU/ml adenosine deaminase and 50 mM Tris-HCl, at pH 7.4. The incubation was started by addition of the membrane suspension (400 microliters of membranes in each tube at a protein concentration of 0.1 mg/ml) to the previous mixture of 0.5 nM [^35^S]GTPγS, 50 μM GDP and appropriate concentrations of drugs and was performed at 30°C for 120 min with shaking. Incubations were terminated by adding 3 ml of ice-cold resuspension buffer followed by rapid filtration through Whatman GF/C filters presoaked in the same buffer. The filters were rinsed twice with ice-cold resuspension buffer, transferred to vials containing 5 ml of OptiPhase HiSafe II cocktail and the radioactivity trapped was determined by liquid scintillation spectrometry (Packard 2200CA). Non-specific binding was defined as the remaining [^35^S]GTPγS binding in the presence of 10 μM unlabelled GTPγS. Non-specific binding was subtracted from total bound radioactivity to determine [^35^S]GTPγS specific binding.

#### Competition Binding Assay

The mixture for the binding assay contained Hanks’ Balanced Salt Solution (HBSS) (NaCl, 8 g/l; CaCl_2_, 0.14 g/l; KCl, 0.40 g/l; NaHCO_3_, 0.35 g/l; glucose, 1 g/l; MgCl_2_ 6H_2_O, 0.10 g/l; KH_2_PO_4_, 0.06 g/l; MgSO_4_ 7H_2_O, 0.10 g/l and Na_2_HPO_4_, 0.05 g/l, pH 7.4), the membrane suspension (2.07 μg of membrane proteins) and 2 nM [^3^H]-CGP54626 with or without different concentrations of baclofen in a final volume of 0.5 ml. The mixture was incubated at 5°C for 20 min, and then the reaction was terminated by adding ice-cold wash buffer followed by rapid filtration through Whatman GF/C filters (presoaked with 0.33% polyethyleneimine with 50 mM Tris-Cl, pH 7.4, for 30 min). The filters were rinsed twice with ice-cold wash buffer, transferred to vials containing 5 ml of OptiPhase HiSafe II cocktail and the radioactivity trapped was determined by liquid scintillation spectrometry (Packard 2200CA).

### Animal Models and Behavioral Tests

Adult male Wistar rats (250–300 g; Charles River France, St Aubin les Elbeuf, France) were used. The experiments followed the ethical guidelines of the International Association for the Study of Pain and were approved by the local ethics committee in Bordeaux (AP 1/04/2005). Persistent neuropathic pain in rats was evoked by ligation of the right L5-L6 spinal nerve (Spinal Nerve Ligation, SNL) (Decosterd and Woolf, 2000). Mechanical response thresholds were monitored in SNL, and sham-operated (sciatic nerve was exposed but the L5 and L6 sciatic nerves were not ligated) animals at 1 day before surgery (reference value for each animal), and at day 12 (before and after intrathecal injections of baclofen) and at day 15 (24 h after the last siRNA injection). Rats were placed in the testing cage 1 h before the test for habituation. The withdrawal threshold of the leg on the operated side was determined in response to mechanical stimuli applied to the plantar surface of the foot. The limb withdrawal threshold was measured by an electronic device (Bioseb, France) that was derived from Von Frey filaments (Kalmar et al., 2003; Lever et al., 2003). Sham-operated rats were used as controls.

### *In vivo* Intrathecal Injections and Mechanical Allodynia

For intrathecal injections of baclofen or RNAs (anti-fibulin-2 siRNA or mismatch RNA), sham and SNL rats were implanted with a catheter (PE-10; Phymep, France) inserted into the subarachnoid space (Fossat et al., 2007). The threshold to mechanical stimulation was monitored before and after surgery, and after the various injections. Results were expressed as percentages of changes in the threshold of neuropathic rats.

#### The Experimental Schedule Is Presented Below


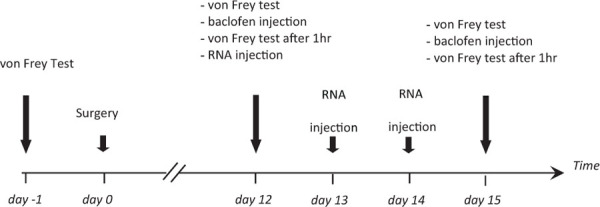


Rats were tested for mechanical allodynia before surgery (day-1, reference value set to 100%). The threshold to mechanical stimulation was monitored again in sham-operated and SNL animals 12 days after surgery (day 12), after baclofen injection (1 μg in 10 μL; Sigma B5399) on the same day (day 12, baclofen), on day 15 after injection of anti-fibulin-2 siRNA or mismatch RNA (day 15, RNA), and finally after baclofen injection on the same day (day 15, RNA + baclofen). Anti-fibulin-2 siRNA (siRNA) and mismatch RNA (mmRNA) were injected daily from day 12 to day 14 according to a published protocol (Luo et al., 2005) and Neuromics instructions (Neuromics, Northfield, MN, United States). In some experiments, saclofen (30 μg, Sigma S166) or CGP55845 (10 μg, Tocris 5584) were co-applied *in vivo* with baclofen.

### Statistical Analysis

Statistical analyses were conducted using SigmaPlot 12.0 software (SigmaStat, Systat Software Inc, San Jose, CA, United States). Results were presented as mean ± standard error of the mean (SEM). The Student’s *t*-test was used for two-sample comparisons. For multiple sample comparisons, one-way ANOVA or two-Way ANOVA followed by a Tukey *post hoc* test was performed for FRET studies. One-way ANOVA followed by a Tukey test was performed for behavioral studies. Regarding the electrophysiology study, for all comparisons of cumulative distributions, 2-sample Kolmogorov–Smirnov test was used. When not indicated otherwise, the Tukey test was used. *p* < 0.05 was considered significant.

## Results

### Fibulin-2 Regulates GABAB Activation *in vitro*

**FIGURE 1:**
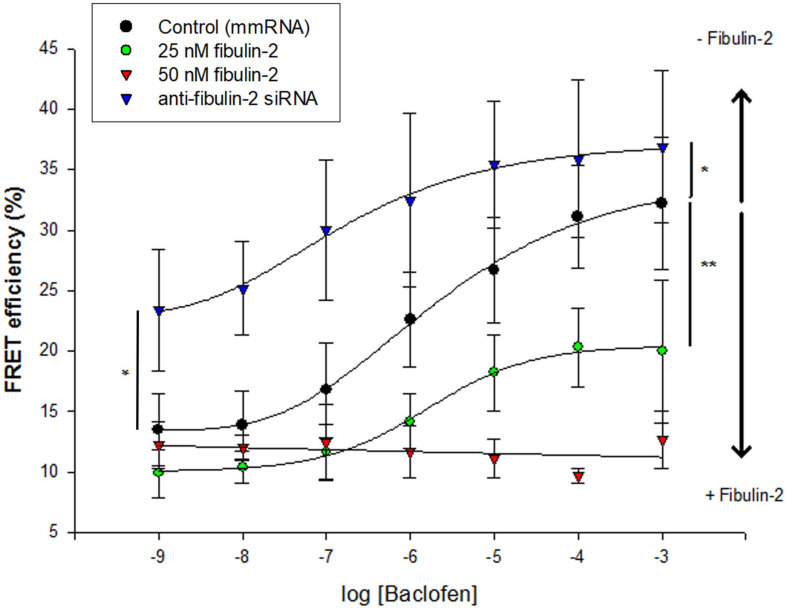
Fibulin-2 regulates baclofen-induced GABAB conformational changes *in vitro.* Fibulin-2 dose-dependently prevents agonist-induced conformational changes. FRET efficiency is measured between GFP-GABAB1a and DsRed-GABAB2 with different concentrations of baclofen (1 nM to 1 mM) (black circles, control). When fibulin-2 expression is inhibited with anti-fibulin-2 siRNA (blue triangles), efficacy of baclofen is increased (maximal effect of blue plot *vs.* black plot, ^∗^*p* < 0.05). In contrast, in cells treated with exogenous fibulin-2 protein at 25 nM (green plot), maximal effect of baclofen is decreased as compared to control (maximal effect of fibulin-2 at 25 nM *vs.* control, ^∗∗^*p* < 0.01). With 50 nM of fibulin-2 (red plot), the FRET efficiency between the two subunits is abolished. 20 < *n* < 30. Two Way ANOVA, Tukey *post hoc* test: ^∗^*p* < 0.05; ^∗∗^*p* < 0.01.

Our first aim was to assess the consequence of GABAB/fibulin-2 interaction on the receptor conformation. We used FRET/FLIM to measure possible changes in the distance or orientation between GFP-GABAB1a and DsRed-GABAB2. Two-way ANOVA revealed that incubation of primary spinal neuronal cultures with fibulin-2, or blocking endogenous fibulin-2 with siRNA, modified baclofen-induced fluorescence resonance energy transfer (FRET) between GABAB1a and GABAB2 receptor subunits [Figure 1; *F*_(3,252)_ = 45.3, *p* < 0.01]. Exogenous fibulin-2 inhibited FRET interaction in a dose-dependent manner. At 25 nM, fibulin-2 decreases the maximal effect (efficacy) of baclofen to induce FRET interactions between B1a and B2 (32.2% ± 5.46 in control *vs.* 20% ± 5.9 in fibulin-2-treated cultures; *p* < 0.01 *vs.* control at 10^–3^M baclofen). At higher doses (50 nM), fibulin-2 totally suppressed FRET interactions. In contrast, siRNA-mediated inhibition of fibulin-2 expression resulted in an increase of baclofen maximal effect as measured with FRET (32.2% ± 5.46 in control *vs.* 36.9% ± 6.3 in siRNA treated cultures, *p* < 0.05 at 10^–3^M baclofen). Interestingly, siRNA treatment also increased the efficacy of low doses of baclofen (13.5% ± 3.02 in control *vs.* 23.4% ± 5 in siRNA treated cultures; *p* < 0.05 *vs.* control at 10^–9^M baclofen).

**FIGURE 2:**
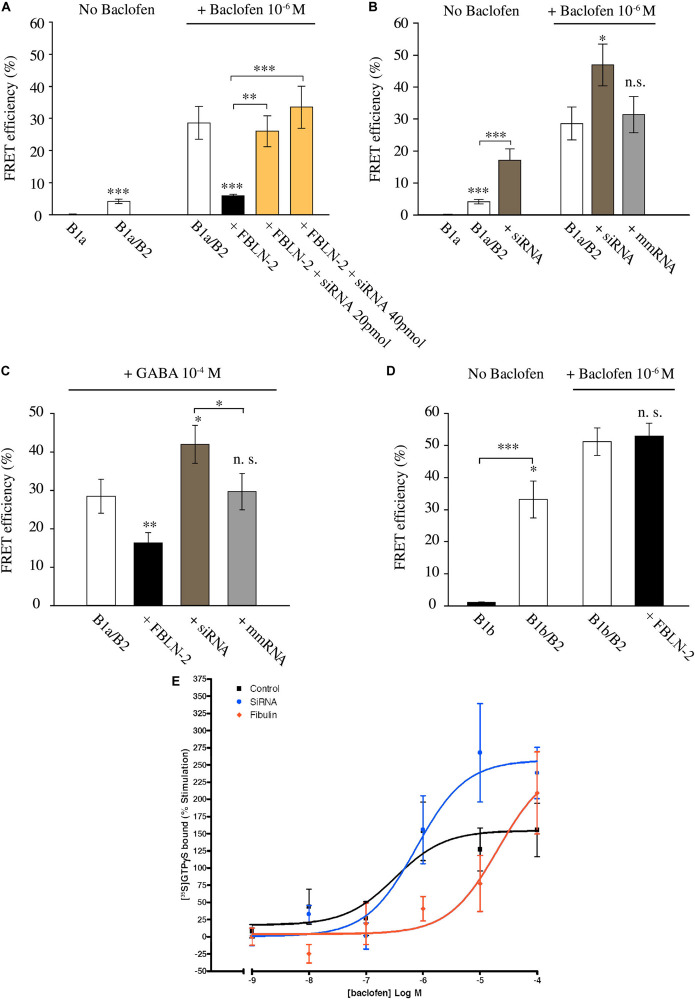
Fibulin-2 regulates agonist-induced GABAB activation *in vitro.* **(A)** FRET efficiency between GFP-GABAB1a and DsRed-GABAB2 ± baclofen at 10^–6^M. FRET efficiency is decreased after overexpression of fibulin-2 and is restored by anti-fibulin-2 siRNA application in a dose-dependent manner (20 or 40 pmol siRNA). ^∗∗∗^*p* < 0.001 *vs.* “B1a/B2 + baclofen” unless indicated; ^∗∗^*p* < 0.01; (*n* = 21 cells). **(B)** FRET efficiency between GFP-GABAB1a and DsRed-GABAB2 ± baclofen at 10^–6^M. FRET efficiency is increased after anti-fibulin-2 siRNA application in basal conditions without baclofen (^∗∗∗^*p* < 0.001 “B1a/B2” *vs.* “B1a/B2 + siRNA”). Agonist-induced receptor activation is increased after transfection with anti-fibulin-2 siRNA but not after transfection with mmRNA (*n* = 21 cells). ^∗^*p* < 0.05; ^∗∗∗^*p* < 0.001; n.s.: *p* > 0.05 *vs.* “B1a/B2 + baclofen” unless indicated. **(C)** FRET efficiency between GFP-GABAB1a and DsRed-GABAB2 ± GABA at 10^–4^M. FRET efficiency is decreased after overexpression of fibulin-2. GABA effect is increased after anti-fibulin-2 siRNA application but not after mmRNA transfection (25 < *n* < 35). ^∗^*p* < 0.05; ^∗∗^*p* < 0.01; n.s.: *p* > 0.05 *vs.* “B1a/B2 + GABA” unless indicated. **(D)** FRET efficiency between GFP-GABAB1b and DsRed-GABAB2 ± baclofen at 10^–6^M. Fibulin-2 overexpression has no effect on the agonist-induced conformational changes of GABAB1b/GABAB2 (16 < n < 31). ^∗^*p* < 0.05; ^∗∗∗^*p* < 0.001; n.s.: *p* > 0.05 *vs.* “B1b/B2 + baclofen” unless indicated. **(E)** Fibulin-2 regulates GABAB signaling *in vitro*. **(A)** Stimulation (% of the basal activity) of [35S]GTPγS binding observed from 1 nM of baclofen to 100 μM. Primary cell cultures of rat cortex treated with anti- fibulin-2 siRNA show higher maximal effect than control cultures (257% ± 29 *vs.* 155% ± 23) but similar EC50. However, fibulin-2 treated cultures show higher EC50 than control cultures (20 *vs.* 0.32 μM). Data are expressed as percentage of stimulation.

Next, we tested for the effectiveness of cell-secreted fibulin-2 to alter GABAB conformation. Fibulin-2-expressing pcDNA plasmid was co-transfected with B1a and B2 subunits, and FRET interactions were measured in spinal cultures as above (Figure 2A). One-way ANOVA indicated that modifying the amount of fibulin-2 in the primary culture has an effect on FRET interactions [Figure 2A, *F*_(3,36)_ = 93.8, *p* < 0.001]. Fibulin-2 overexpression decreased baclofen-stimulated FRET interactions (Figure 2A, 28.6% ± 5.11 in B1a/B2 *vs.* 5.9% ± 0.4 in B1a/B2+fibulin-2 transfected cultures; *p* < 0.001). Moreover, after fibulin-2 overexpression, baclofen-induced FRET interactions remained in the same range as constitutive FRET interactions, without baclofen, indicating a nearly total suppression of baclofen effects. Fibulin-2-induced inhibition of FRET interactions was countered by anti-fibulin-2 siRNA application, in a dose-dependent manner (Figure 2A, 20 or 40 pmol anti-fibulin-2 siRNA).

Since various cell types normally secrete fibulin-2 in the extracellular matrix, we investigated the possible role of endogenous fibulin-2 in restraining GABAB conformational changes. For this purpose, we incubated spinal cultures with anti-fibulin-2 siRNA (without overexpressing fibulin-2) (Figure 2B). When fibulin-2 endogenous expression was blocked with siRNA, FRET interactions were increased in both unstimulated and baclofen-activated GABAB receptor. In contrast, mismatch RNA had no effects on energy transfer between GABAB subunits.

Then, we tested whether fibulin-2 was also able to inhibit the effects of GABA, the endogenous GABAB ligand (Figure 2C). As for baclofen, we found that fibulin-2 overexpression decreased GABA-stimulated FRET interactions (Figure 2C, 28.5% ± 4.4 in B1a/B2 *vs.* 16.3% ± 2.7 in cultures overexpressing fibulin-2; *p* < 0.01). Moreover, anti-fibulin-2 siRNA potentiates the effects of GABA (Figure 2C, 28.5% ± 4.4 in B1a/B2 *vs.* 42% ± 4.9 in B1a/B2+siRNA *vs.* B1a/B2; *p* < 0.05) presumably by suppressing inhibition of receptor activation by endogenous fibulin-2.

We also checked the specificity of fibulin-2 action on the B1/B2 heterodimer subtype. Fibulin-2 had no effect on baclofen-stimulated FRET interactions between B2 subunit and B1b, the other transcript variant of B1 subunit (Figure 2D; 51.2% ± 4.3 in B1b/B2 *vs.* 53% ± 3.9 in B1b/B2 + fibulin-2 transfected cultures, *p* > 0.05, *t*-test). This was expected since B1b does not contain the extracellular Sushi domains that bind fibulin-2 (Blein et al., 2004).

Finally, GTP binding assays were performed on membrane extracts from cortical cell cultures (Figure 2E). In normal conditions, baclofen facilitated G-protein coupling dose-dependently, with a maximum effect of 155 ± 23% of stimulation over basal values and micromolar potency (logEC50 = −6.5 ± 0.45 μM). Fibulin-2 application decreased the potency of baclofen on G-protein activation by two orders of magnitude (logEC50 = −4.7 ± 0.46 μM). The increase in the siRNA group reached up to a 100% of further stimulation by baclofen when the maximal effect was compared with the control group. In this siRNA group, G-protein coupling efficacy reached a maximum effect of 257 ± 29% and a similar potency than control group (logEC50 = −6.1 ± 0.29 μM).

Taken together, these data show that fibulin-2 hampers conformational changes of the GABAB receptor, both constitutively and upon agonist application. These changes in the GABAB conformation correlate with fibulin-2-induced loss in G-protein coupling. Finally, we showed with a siRNA-based strategy that endogenous fibulin-2 exerts a tonic inhibitory effect on GABAB activation as revealed by FRET analysis and GTP binding assay.

### Mechanism of Fibulin-2 Action

**FIGURE 3:**
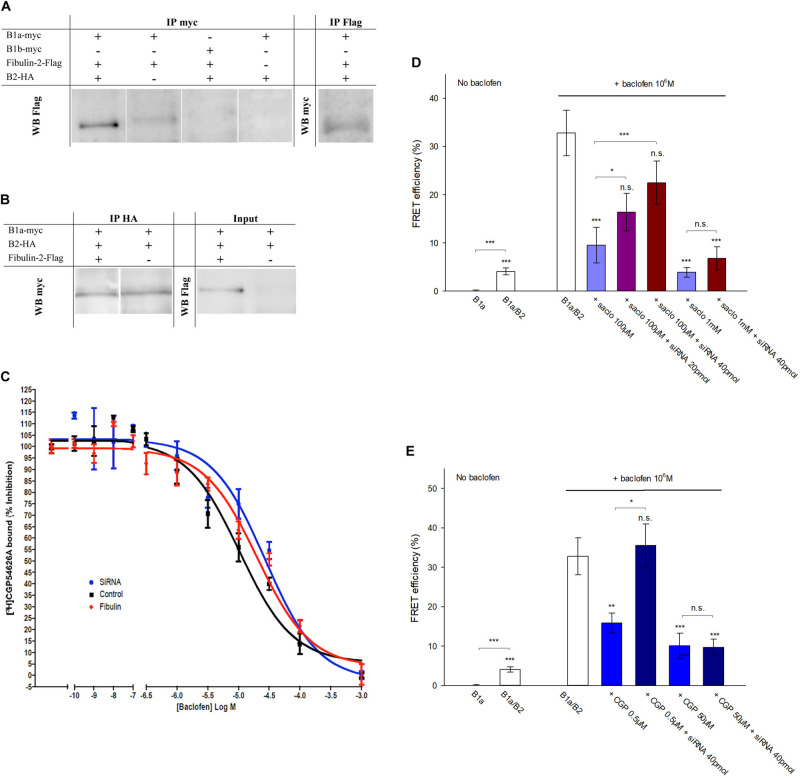
Fibulin-2 interacts with GABAB and alters antagonist-induced effects *in vitro*. **(A)** Fibulin-2-Flag co-immunoprecipitates with myc-GABAB1a (lane 1, left panel) in COS-7 cells. In the absence of HA-GABAB2, the co-immunoprecipitation is largely reduced (lane 2, left panel). Fibulin-2-Flag does not co-immunoprecipitate with the GABAB1b subunit (lane 3, left panel). No signal is observed in the absence of myc-GABAB1a or fibulin-2-Flag (lane 3 and 4, left panel). Conversely, myc-GABAB1a co-immunoprecipitates with fibulin-2-Flag (right panel). IP, immunoprecipitation; WB, Western-Blot. **(B)** GABAB1a-GFP subunit co-immunoprecipitates with HA-GABAB2 in COS-7 cells in the presence (lane 1, left panel) or in the absence (lane 2, left panel) of fibulin-2-Flag. Fibulin-2-flag transfection actually results in the expression of the protein (right panel). IP: immunoprecipitation; WB: Western-Blot. **(C)** One site fitting of the [^3^H]-CGP54626 binding sites (GABAB receptor) inhibited by baclofen in rat primary cell cultures (expressed in % of inhibition). The membrane preparations from cell cultures were incubated with 2 nM [^3^H]-CGP54626 and increasing concentrations of baclofen. The affinity of the GABAB receptor to the specific agonist baclofen was similar with fibulin-2 (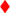); anti-fibulin-2 siRNA (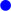) controls (■). pIC50_control_: –5.0 ± 0.1; pIC50_siRNA_: –4.6 ± 0.1; pIC50_fibulin_: –4.7 ± 0.1. **(D)** Effects of saclofen on FRET efficiency between GFP-GABAB1a and DsRed-GABAB2 ± baclofen at 10^–6^M. Baclofen effect is reversed by saclofen at 100 μM and 1 mM. Application of anti-fibulin-2 siRNA (20 or 40 pmol) suppresses this reversion at 100 μM dose (***p < 0.001 “B1a/B2 + saclo100 μM” *vs.* “B1a/B2 + saclo100 μM + siRNA40”). At high dose (1 mM), saclofen remains efficient even in the presence of anti-fibulin-2 siRNA (NS: *p* > 0.05 “B1a/B2 + saclo1 mM” *vs.* “B1a/B2 + saclo1 mM + siRNA40”) (*n* = 21 cells). *p < 0.05; **p < 0.01; ***p < 0.001; n.s.: *p* > 0.05 *vs.* “B1a/B2 + baclofen” unless indicated. **(E)** Effects of CGP55845 on FRET efficiency between GFP-GABAB1a and DsRed-GABAB2 ± baclofen at 10^–6^M. Baclofen effect is reversed by CGP55845 at 0.5 and 50 μM. Application of anti-fibulin-2 siRNA (40 pmol) suppresses this reversion at 0.5 μM dose (*p < 0.05 “B1a/B2 + CGP 0.5 μM” *vs.* “B1a/B2 + CGP 0.5 μM + siRNA40”). At high dose (50 μM), CGP remains efficient even in the presence of anti-fibulin-2 siRNA (NS: *p* > 0.05 “B1a/B2 + CGP50 μM” *vs.* “B1a/B2 + CGP50 μM + siRNA40”) (*n* = 21 cells). *p < 0.05; **p < 0.01; ***p < 0.001; n.s.: *p* > 0.05 *vs.* “B1a/B2 + baclofen” unless indicated.

With regards to the mechanisms accounting for fibulin-2-induced impairment of GABAB activation, we first confirmed the interaction of the ECM protein with the GABAB receptor. Our attempts to perform co-immunoprecipitation of endogenous fibulin-2 and GABAB receptor subunits failed and the detection of endogenous fibulin-2 in western blot remained below the level of sensitivity, probably due to technical issues with immunoblotting of fibulin-2. We then switched to a cell culture approach and performed immunoprecipitation after transfection of tagged-proteins in COS-7 cells. Our study demonstrated that co-immunoprecipitation of myc-GABAB1a with an anti-myc antibody retained fibulin-2-Flag (Figure 3A, left panel) in cultures that were triple transfected with myc-GABAB1a, HA-GABAB2, and fibulin-2-Flag. The reverse experiments indicated that fibulin-2-Flag also precipitated myc-GABAB1a in the same culture model (Figure 3A, right panel). Interestingly, the co-immunoprecipitation of myc-GABAB1a and fibulin-2-Flag was partly lost in double transfected cell cultures, lacking the GABAB2-expressing plasmid (Figure 3A). This suggested that most of myc-GABAB1a was unable to bind fibulin-2-Flag, most probably because it was not exposed to the plasma membrane in this condition, remaining trapped in the endoplasmic reticulum due to the relatively low amount of endogenous GABAB2 that normally ensures proper trafficking of the receptor (White et al., 1998; Kuner et al., 1999; Margeta-Mitrovic et al., 2000). A control experiment was conducted in cell cultures triple transfected with myc-GABAB1b, GABAB2 and fibulin-2-Flag (Figure 3A). No co-immunoprecipitation between myc-GABAB1b and fibulin-2-Flag was detected in agreement with the lack of fibulin-2-induced changes in GABAB1b/GABAB2 FRET interactions (see Figure 2D). This confirmed that fibulin-2 interacts with GABAB1a but not GABAB1b which lacks the fibulin-2-interacting sushi-domain.

We then considered that fibulin-2 may trigger a possible loss of the heterodimeric state of the GABAB receptor as previously demonstrated for the B1b/B2 post-synaptic subtype (Laffray et al., 2007). Indeed, heterodimer dissociation would lead to similar changes in FRET interactions and GTP binding activity. We tested this hypothesis with co-immunoprecipitation of myc-GABAB1a and HA-GABAB2 subunits transfected in COS-7 cells in the presence or in the absence of fibulin-2 (Figure 3B). No differences could be shown in B1a/B2 association in the different conditions tested, whereby ruling out the possibility that fibulin-2 alters the dimeric state of the GABAB receptor.

Hence, we hypothesized that fibulin-2 could modify GABAB pharmacology rather than disrupting the dimeric state. We first tested the hypothesis that fibulin-2 modifies the affinity of the agonist for the GABAB receptor. For this purpose, we performed binding experiments to displace [^3^H]-CGP54626 by increasing concentration of baclofen after application of exogenous fibulin-2 or anti-fibulin-2 siRNA (Figure 3C). No effect was seen regarding the IC50 by baclofen. These data suggest that fibulin-2 induces effects on GABAB receptors in a more mechanical way, i.e., hampering conformational changes of the receptor. Then, we explored changes in pharmacological properties by studying with FRET the efficiency of GABAB antagonists to revert baclofen activation in control conditions, or in the absence of fibulin-2 (Figures 3D,E). As expected, both saclofen (100 μM, Figure 3D) and CGP55845 (0.5 μM, Figure 3E) efficiently countered baclofen-stimulated GABAB activation. Interestingly, this antagonistic effect was abolished when cultures were treated with anti-fibulin-2 siRNA. Higher concentrations of saclofen (1 mM) or CGP55845 (50 μM) were able to restore the antagonist efficiency, showing that fibulin-2 alters pharmacological properties, i.e., antagonist sensitivity in presence of baclofen, of the B1a/B2 receptor subtype.

### Functional Effects of Fibulin-2 on Inhibitory Transmission

**FIGURE 4:**
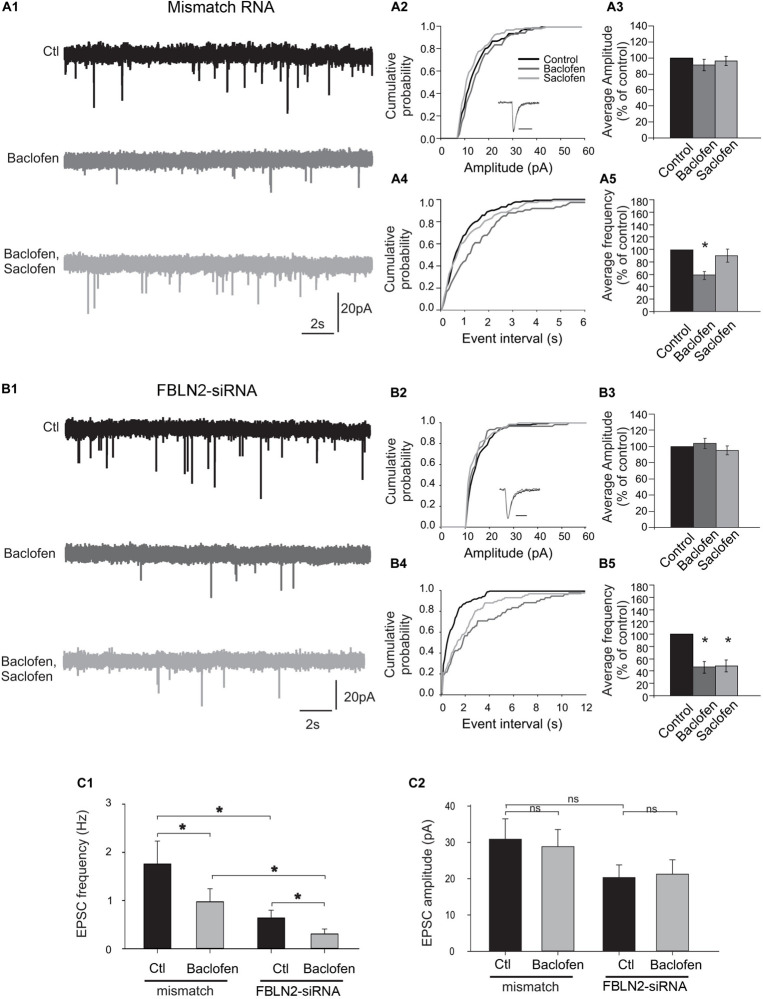
Fibulin-2 controls the sensitivity to baclofen and saclofen of GABAB-mediated presynaptic inhibition. **(A1)** Spontaneous miniature EPSCs (mEPSCs) recorded from cultured spinal neurons transfected with mismatch siRNA before and after application of baclofen (10 μM) and baclofen + saclofen (100 μM). (**A2)** Amplitude cumulative probability for the experiment shown in **(A1)**. Inset trace shows superimposed average of 25 mEPSCs obtained in control and in presence of baclofen. **(A3)** Inter-event interval cumulative probability for the experiment shown in **(A1)**. The distribution is significantly shifted toward the right during baclofen application. **(A4,A5)** average variation in mEPSC amplitude **(A4)** and frequency **(A5)**, expressed as percent of control (*n* = 9). **(B1)** mEPSCs recorded from cultured spinal neurons transfected with fibulin-2 siRNA before and after application of baclofen (10 μM), saclofen (100 μM), and CGP (50 μM). **(B2)** Amplitude cumulative probability for the experiment shown in **(B1)**. Inset trace shows superimposed average of 25 mEPSCs obtained in control and in presence of baclofen. **(B3)** Inter-event interval cumulative probability for the experiment shown in **(B1)**. The distribution is shifted toward the right during baclofen and baclofen + saclofen application. **(B4,B5)** Average variation in mEPSC amplitude **(B4)** and frequency **(B5)**, expressed as percent of control (*n* = 7). **(C1,C2)** Average frequencies **(C1)** and amplitudes **(C2)** of mEPSCs recorded in preparations transfected with mismatch (*n* = 9) or anti fibulin-2 siRNA (*n* = 7), in control conditions and in the presence of baclofen (10 μM).

Then, we asked whether fibulin-2 modifies B1a/B2-mediated inhibition. Since the B1a subunit is mainly found in presynaptic compartments (Vigot et al., 2006) we focused on presynaptic GABAB inhibition on spinal neuron cultures (Figure 4). To further investigate the role of fibulin-2 in the control of synaptic transmission, we analyzed the effects of baclofen (10 μM) and saclofen (100 μM) on the amplitude and frequency distributions of miniature excitatory EPSCs recorded in the presence of TTX (1 μM). In preparations transfected with mismatch RNA (Figure 4A1), comparison of the mEPSC amplitude distribution (Figure 4A2) revealed no significant change between control and baclofen conditions (Kolmogorov–Smirnov test, KS). Moreover, the kinetics of averaged mEPSCs was not affected by baclofen application (see inset in Figure 4A2). Overall, in 9 recorded neurons, the average mEPSC amplitude remained unaltered by baclofen application (Figure 4A3, paired *t*-test). By contrast, the inter-event interval was significantly shifted toward the right (Figure 4A4), indicating a decrease in mEPSC frequency (KS test, *p* < 0.001, *n* = 9). Further saclofen addition (100 μM), in the continuous presence of baclofen, induced a recovery of mEPSCs frequency in 7 out of 9 recorded cells. Overall (Figure 4A5), baclofen induced a significant decrease in mEPSCs frequency to 58.2% ± 6.6 of control (*p* < 0.05, paired *t*-test). Whereas in the presence of saclofen, the average mEPSC frequency returned to control value (resp 91% ± 9). These results demonstrate a clear presynaptic inhibition of excitatory synaptic transmission in cultured spinal neurons. Similar results were obtained in control preparations as well as preparations exposed to transfecting agent alone (*n* = 9 and *n* = 7 respectively, data not shown).

In preparations transfected with fibulin-2 siRNA (Figure 4B1), baclofen had no effect on the amplitude distribution (Figure 4B2, KS test), and the kinetics of averaged mEPSCs remained unchanged (inset in B2). No statistical effect of baclofen on average mEPSCs amplitude could be observed (Figure 4B3, paired *t*-test, *n* = 7). Baclofen induced a shift in the frequency distribution curve (Figure 4B4, *p* < 0.001), indicating a clear presynaptic inhibition of mEPSCs. However, in contrast with preparations transfected with mismatch siRNA, further saclofen application (100 μM) failed to antagonize baclofen effects (Figure 4B2, KS test), on mEPSCs frequency. Overall (Figure 4B5), baclofen application induced a decrease in mEPSCs frequency to 44.4 ± 11.4% of control values in 7 out of 7 cells (paired *t*-test, *p* < 0.05). In 5 out of these 7 neurons, further application of saclofen did not alter mEPSC frequency (54 ± 0.18% of control, paired-*t*-test, *p* < 0.05), showing that the antagonist loses its capacity to block presynaptic baclofen-induced activation of the GABAB receptor at the dose used when fibulin-2 is knocked-down.

Finally, in preparations transfected with fibulin-2 siRNA, the average mEPSC frequency was significantly lower than in preparations transfected with mismatch RNA (Figure 4C1). Importantly, this effect was preserved in the presence of baclofen (Figure 4C1). This result indicates that the absence of fibulin-2 facilitates the action of baclofen and the presynaptic inhibitory effect of GABAB signaling. In contrast, no change was observed in the mean mEPSC amplitude (Figure 4C2).

### Expression of Fibulin-2 *in vivo*

**FIGURE 5:**
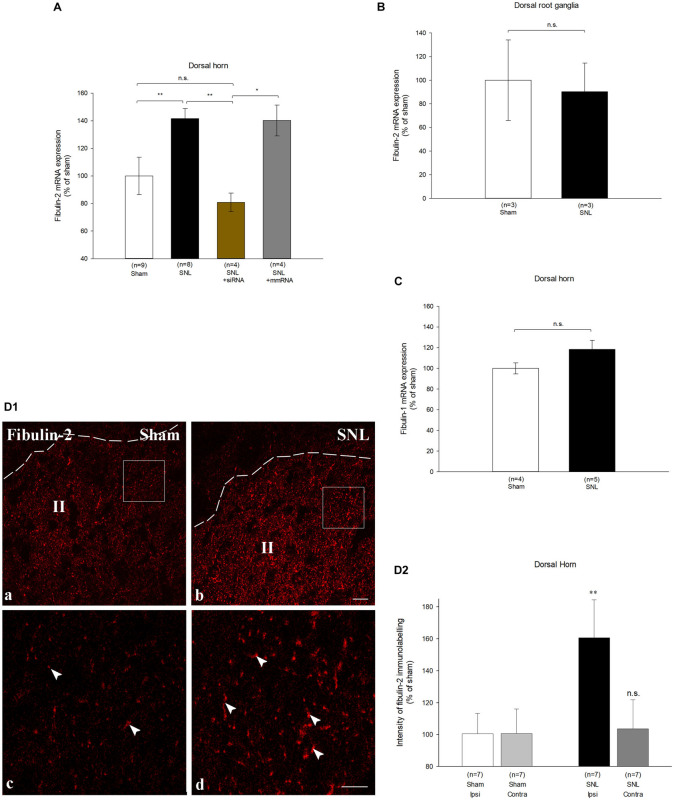
Fibulin-2 is upregulated in the spinal cord of neuropathic rats. **(A)** qRT-PCR analysis of fibulin-2 mRNA in the ipsilateral spinal dorsal horn of sham rats, neuropathic rats (SNL), and SNL rats injected with anti-fibulin-2 siRNA (SNL + siRNA) or with mismatch RNA (SNL + mmRNA). Data are expressed as percentage of sham ± SEM. Fibulin-2 expression increased in ipsilateral dorsal horn of neuropathic rats. This increase was efficiently prevented by siRNA injection. The injection of mmRNA had no effect on fibulin-2 expression. *p < 0.05, **p < 0.01 “sham” *vs.* “SNL”; n.s.: *p* > 0.05. **(B)** qRT-PCR analysis of mRNA of fibulin-2 in the ipsilateral dorsal root ganglia of sham and neuropathic (SNL) rats. Data are expressed as percentage of sham ± SEM. No significant changes are seen (n.s.: *p* > 0.05 “sham” *vs.* “SNL). **(C)** qRT-PCR analysis of mRNA of fibulin-1 in the ipsilateral spinal dorsal horn of sham and neuropathic (SNL) rats. Data are expressed as percentage of sham ± SEM. No significant changes are observed (n.s.: *p* > 0.05 “sham” *vs.* “SNL). **(D1)** Immunohistochemistry for endogenous fibulin-2 in the ipsilateral dorsal horn of sham **(a)**, and neuropathic (SNL) **(b)** rats. The dotted line indicates the dorsal limit of the spinal cord section. Framed areas of the lamina II (II) in **(a,b)** are displayed at higher magnification in **(c,d)**, respectively. Fibulin-2 expression was very low in sham animals (arrowheads in **c**) whereas an intense staining could be seen in the SNL group (arrowheads in **d**). **(D2)** The quantification of the signal intensity confirmed the significant difference in the ipsilateral dorsal horn between sham and SNL groups. In contrast, no changes is seen in the contralateral dorsal horn (*n* = 3 sections from 7 animals in sham and SNL groups). **p < 0.01; n.s.: *p* > 0.05.

We next investigated whether changes in fibulin-2 expression can alter GABAB inhibition *in vivo*. Since GABAB is involved in the modulation of nociceptive transmission (Bowery, 2006) we studied fibulin-2 expression in rats with spinal nerve ligation (SNL) that induced neuropathic pain. Real-time RT-PCR showed a marked increase in fibulin-2 mRNA levels in the dorsal spinal cord of SNL rats ipsilateral to the injury, as compared to sham animals (Figure 5A; One-way ANOVA, *F*_(3,21)_ = 11.67, *p* < 0.01; 141.58% ± 7.2 in SNL rats; *p* < 0.01 *vs.* sham). Fibulin-2 overexpression was limited to the spinal cord since no increase was noticed in the dorsal root ganglia (Figure 5B). However, the spinal cell types that secretes fibulin-2 remain to establish. Overexpression was specific of fibulin-2 since the expression of the related fibulin-1 showed no changes (Figure 5C).

We checked fibulin-2 upregulation at the protein level with immunohistochemistry. Immunostaining was hardly detectable in control animal dorsal horn (Figure 5D1, left). Intensity was much higher in SNL rats (Figure 5D1, right) where discrete staining was observed. Changes in immunostaining intensity were quantified and showed a 60% increase in the ipsilateral dorsal horn of SNL rats as compared to sham animals (Figure 5D2; 160.7% ± 23.7 of the sham intensity; *p* < 0.01, *t*-test). No changes were detected in the contralateral dorsal horn (Figure 5D2), or in the ventral horn (data not shown).

**FIGURE 6:**
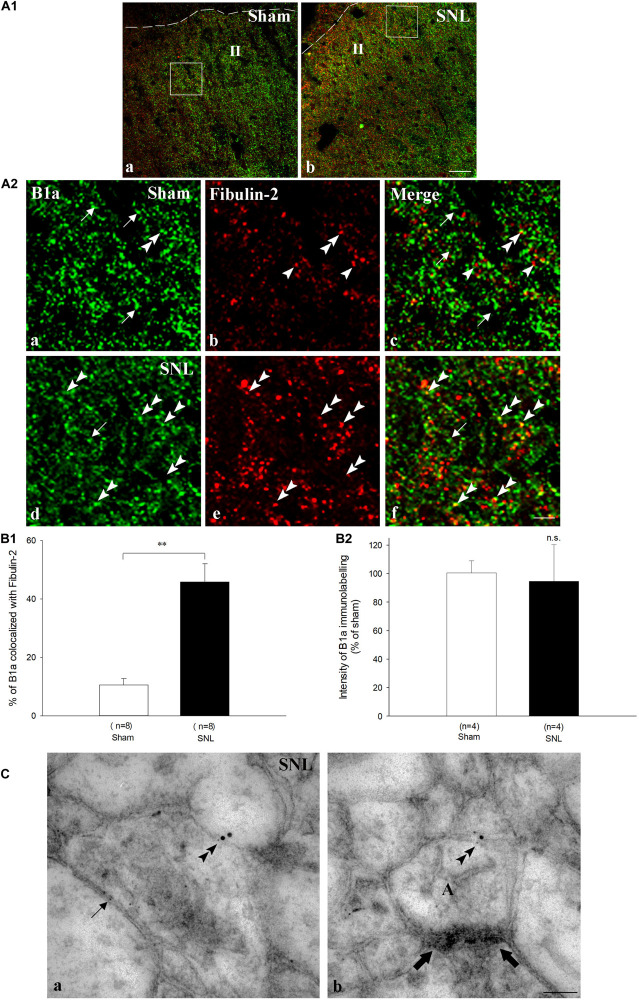
Endogenous co-expression of B1a and fibulin-2 in sham and neuropathic rats. **(A1)** Immunohistochemistry for endogenous B1a (green) and fibulin-2 (red) in lamina II (II) of the dorsal horn of sham **(a)**, and neuropathic (SNL) **(b)** rats. The dotted line indicates the dorsal limit of the spinal cord section. **(A2)** Micrographs are higher magnification of the framed areas in **(A1)** and correspond to sham **(a–c)**, and SNL **(d–f)** rats. In sham animals, B1a subunit (green, arrows) and fibulin-2 (red, arrowheads) are distributed throughout the dorsal horn but show very little colocalization. In contrast, colocalization was more frequent in SNL rats (double arrowheads). However, some single labeling was also found for B1a (arrow) and fibulin-2. Bar = 15 μm (same for all). **(B)** Quantification of colocalization in the ipsilateral dorsal horn, expressed as the percentage of B1a labeling colocalized with fibulin-2 (*n* = 8 sections from 3 animals in the sham group; *n* = 7 sections from 3 animals in the SNL group). ^∗∗^*p* < 0.01. **(C)** Double immunogold labeling for B1a (15 nm colloidal gold diameter) and fibulin-2 (5 nm colloidal gold diameter) in the ipsilateral dorsal horn of SNL rats. Both proteins colocalized at the plasma membrane of spinal neurons (double arrowheads in **a,b**). Gold particles association was found essentially at extra-synaptic sites (**b**, the synapse is indicated with large arrows). Single fibulin-2 labeling was also found in the vicinity of the plasma membrane (**a**, arrow). A, axonal nerve ending; bar = 100 nm.

**FIGURE 7:**
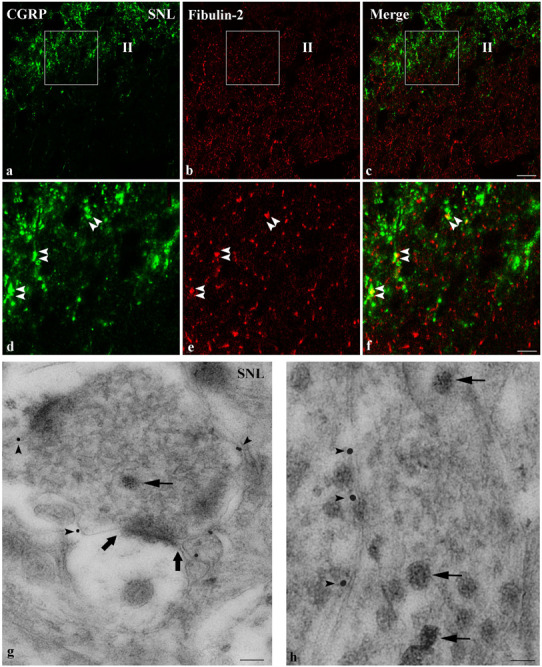
Endogenous co-expression of fibulin-2 and CGRP in neuropathic rats. **(a–f)** Immunohistochemistry for endogenous fibulin-2 and CGRP in lamina II of the dorsal horn of neuropathic **(a–f)** rats. Framed areas of the lamina II (II) in a to c are displayed at higher magnification in **(d–f)**, respectively. CGRP (green) and fibulin-2 (red) staining was frequently seen overlapping or in close apposition (double arrowheads). Bar = 15 μm (same for all). **(g,h)** Double immunogold labeling for fibulin-2 (15 nm colloidal gold diameter) and CGRP (5 nm colloidal gold diameter) in the ipsilateral dorsal horn of neuropathic rats. CGRP was found in secretory granules (**d,e**, arrows). Secretory granules-containing processes were surrounded by fibulin-2 labeling, at the plasma membrane (**d,e**, arrowheads), mostly at extra-synaptic sites (large arrows in **d**). Bar = 100 nm in **(d)**; 50 nm in **(e)**.

We further compared the distribution of fibulin-2 with B1a subunit (Figure 6), and CGRP (Figure 7), a marker of peptidergic nociceptive inputs with light and electron microscopy double labeling experiments.

In sham animals, colocalization of fibulin-2 and B1a appeared scarce, mainly due to the low expression level of fibulin-2 (Figures 6A1,A2a–c). Quantitative analysis confirmed that the rate of B1a colocalization with fibulin-2 remained very limited (Figure 6B1; 10.6 % ± 2.2 of B1a labeled area colocalized with fibulin-2). In SNL rats, fibulin-2 was seen as discrete, intense staining, intermingled with B1a labeling (Figures 6A1,A2d–f). Positive signal was found surrounding cell bodies and in processes. Overlap was much more abundant than in sham animals (Figure 6B1; 45.8% ± 6.2 of B1a labeled area colocalized with fibulin-2 in SNL rats, *p* < 0.01 *vs.* sham rats, *t*-test). However, B1a singly labeled structures were also frequent, suggesting that B1a/fibulin-2 interaction does occur only for a subpopulation of B1a/B2 receptors. No changes in the B1a labeling intensity were detected (Figure 6B2). The increased colocalization is thus likely due to the sole up-regulation of fibulin-2 overexpression. Electron microscopy further demonstrated that B1a and fibulin-2 colocalized on neuronal plasma membrane (Figure 6C). Gold particle association was found only at extra-synaptic sites. Again, both B1a and fibulin-2 labeling were also found independently, as single labeling, both intracellularly and at the membrane.

Regarding fibulin-2/CGRP double labeling, both signals were seen most often in close vicinity with light microscopy (Figures 7a–f). Electron microscopy confirmed that fibulin-2 is localized in the vicinity of the plasma membrane, around sensory nerve endings filled with CGRP-containing secretory granules (Figures 7g,h). These data suggested fibulin-2 interaction with GABAB receptors is located, at least partly, on nociceptive nerve endings in the spinal dorsal horn.

### Functional Role of Fibulin-2 on Neuropathic Pain Sensitization

**FIGURE 8:**
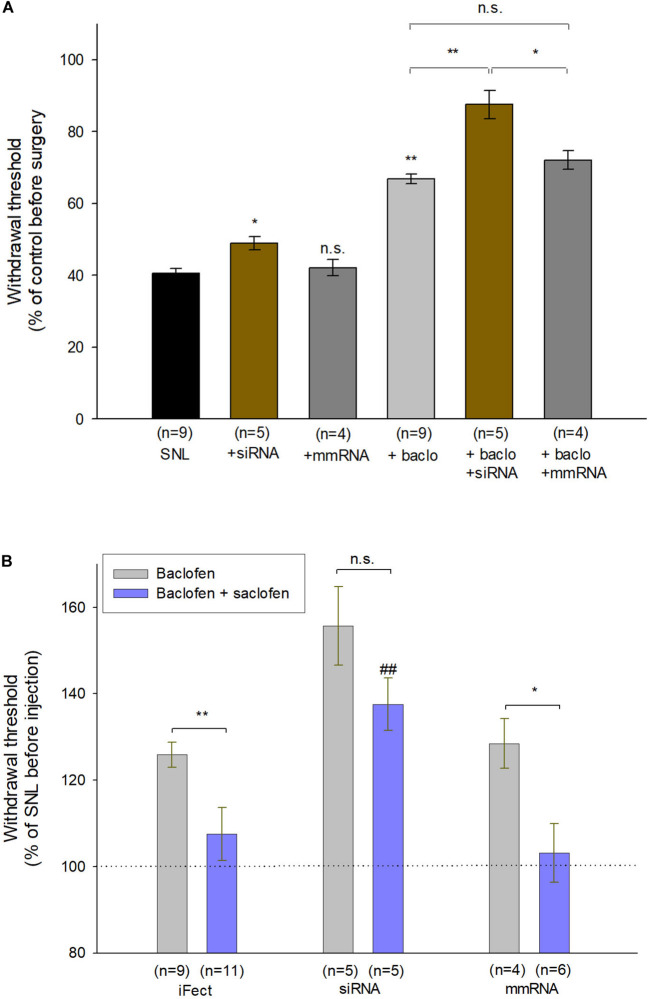
Intrathecal injection of anti-fibulin-2 siRNA potentiates the antinociceptive effect of baclofen in neuropathic rats. **(A)** Effects of anti-fibulin-2 siRNA and baclofen injections in SNL rats. The effects are quantified with or without baclofen, and before (SNL, SNL + baclo) or after (SNL + siRNA, SNL + baclo + siRNA) intrathecal injections of anti-fibulin-2 siRNA or mismatch RNA (SNL + mmRNA, SNL + baclo + mmRNA). Threshold to mechanical stimulation was measured with the von Frey test. In the absence of baclofen, the injection of anti-fibulin-2 siRNA, but not mmRNA, induces an analgesic effect *per se* (*p < 0.05 “SNL” *vs.* “SNL + siRNA”). Baclofen displayed an antinociceptive effect in SNL rats (**p < 0.01 “SNL” *vs.* “SNL + baclo”). Anti-fibulin-2 siRNA potentiated the antinociceptive effect of baclofen in SNL rats (**p < 0.01 “SNL + Baclo” *vs.* “SNL + baclo + siRNA”). The withdrawal threshold remained unchanged after injection of mismatch RNA. The withdrawal threshold before nerve ligation was set to 100%. *p < 0.05; **p < 0.01; n.s.: *p* > 0.05. **(B)** Effects of baclofen and saclofen co-application after injections of iFect (control condition), anti-fibulin-2 siRNA (siRNA) or mismatch RNA (mmRNA) in neuropathic rats. Saclofen inhibited the baclofen effect before anti-fibulin-2 siRNA injection (**p < 0.01 “baclofen” *vs.* “baclofen + saclofen”). This effect of saclofen is abolished after anti-fibulin-2 siRNA injections (n.s.: *p* > 0.05, siRNA, “baclofen” *vs.* “baclofen + saclofen”) but not after mmRNA injection (*p < 0.05, mmRNA, “baclofen” *vs.* “baclofen + saclofen”). After anti-fibulin-2 siRNA injection, the withdrawal threshold was significantly higher than after iFect injection (##*p* < 0.01 siRNA “baclofen + saclofen” *vs.* iFect “baclofen + saclofen”). The withdrawal threshold in neuropathic rats before injection was set to 100%.

Finally, to assess the functional role of fibulin-2 on GABAB inhibition in chronic pain conditions, we tested the behavioral effect of anti-fibulin-2 siRNA intrathecal injection in SNL neuropathic rats (Figure 8). Using the von Frey test, we compared the effects of topical intrathecal application of baclofen and GABAB antagonist on paw withdrawal threshold following intrathecal treatment with anti-fibulin-2 siRNA or mismatch RNA.

One-way ANOVA indicated an effect on the withdrawal threshold [Figure 8A; *F*_(5,30)_ = 32.85, *p* < 0.01]. In the absence of baclofen, anti-fibulin-2 siRNA produces a slight, though significant, analgesic effect (Figure 8A; 40.4% ± 1.4 in SNL *vs.* 48.9% ± 1.9 in SNL + siRNA; *p* < 0.05). The injection of mmRNA does not significantly affect the withdrawal threshold (Figure 8A; 40.4% ± 1.4 in SNL *vs.* 42.1% ± 2.3 in SNL + mmRNA; *p* < 0.05). Interestingly, the baclofen treatments enhance the effects of anti-fibulin-2 siRNA. More particularly, *post hoc* analysis shows that the baclofen plus siRNA treatment is more efficient than baclofen alone, or baclofen plus mmRNA to alleviate pain. Surgery resulted in a ∼60% decrease in paw withdrawal threshold (Figure 8A). The injection of vehicle (iFect) after surgery did not further change the mechanical threshold of neuropathic rats (data not shown). The injection of baclofen at day 12 after surgery induced an increase in mechanical threshold indicating GABAB-mediated antinociceptive effects (Figure 8A; 40.4% ± 1.4 in SNL *vs.* 66.8% ± 1.4 in SNL + baclo; *p* < 0.01). This response to baclofen was significantly increased in SNL rats treated with anti-fibulin-2 siRNA (Figure 8A; 66.8% ± 1.4 in SNL + baclo *vs.* 87.5% ± 3.9 in SNL siRNA + baclo, *p* < 0.01). In contrast, the mean threshold for SNL animals was significantly increased upon siRNA treatment after baclofen application. Changes in response to baclofen were not observed in animals injected with mmRNA (Figure 8A; 66.8% ± 1.4 in SNL + baclo *vs.* 72.0% ± 2.6 in SNL mmRNA + baclo, *p* > 0.05) showing paw withdrawal threshold similar to those observed in neuropathic rats before treatment.

The co-injection of baclofen and saclofen, in SNL rats, reversed the effects of baclofen alone (Figure 8B; 128.12% ± 2.62 in baclofen iFect *vs.* 108.79% ± 7.3 in baclofen plus saclofen iFect, *p* < 0.01). In the baclofen plus saclofen iFect group, paw withdrawal threshold was similar to that measured in SNL rats without injection, indicating the persistence of mechanical allodynia. In contrast, after anti-fibulin-2 siRNA application, saclofen was unable to significantly block baclofen effects. The increase of paw withdrawal threshold remained statistically unchanged (Figure 8B; 155.7% ± 9.4 in baclofen siRNA *vs.* 142.56% ± 6 in baclofen plus saclofen siRNA, *p* > 0.05). In both “baclofen” and “baclofen plus saclofen” groups, the paw withdrawal threshold was higher than after the control iFect injection. The injection of mmRNA did not change the effects of baclofen alone, or baclofen plus saclofen injection, and the withdrawal threshold remained different between the two groups (Figure 8B; 128.5% ± 5.75 in baclofen mmRNA *vs*. 103.2% ± 6.7 in baclofen plus saclofen mmRNA; p< 0.05). These data demonstrate that fibulin-2 limits GABAB receptor inhibition *in vivo*. Knocking down fibulin-2 *in vivo* facilitates conformational changes and activation of the receptor, therefore enhancing GABAB-induced alleviation of pain.

## Discussion

In the present study, we demonstrated that: (1) fibulin-2 decreased the agonist-induced activation of GABAB receptor; (2) fibulin-2 modified the receptor sensitivity to antagonists; (3) fibulin-2 regulated the GABAB-mediated inhibition of neurotransmitter release; (4) fibulin-2 was up-regulated in rat models of neuropathic pain; (5) intrathecal knock-down of fibulin-2 expression partially reversed mechanical allodynia in neuropathic rats.

### GABAB1a Interaction With Fibulin-2

GABAB interacting proteins were demonstrated to control several aspects of the receptor functions (reviewed in Xu et al., 2014) including receptor dimerization [14.3.3ζ (Couve et al., 2001; Laffray et al., 2012)], intracellular targeting [MUPP1 (Balasubramanian et al., 2007), CHOP (Sauter et al., 2005)], signaling transduction and desensitization [ATF4 (Vernon et al., 2001), GRK4 (Perroy et al., 2003)]. Only few interactions between partner proteins and the GABAB1a sushi domains have been described. GABAB1a sushi domains play important roles in receptor trafficking by stabilizing the GABAB1a/GABAB2 dimer at the cell surface (Biermann et al., 2010; Hannan et al., 2012). But the possibility that the sushi domains contribute to signaling transduction and physiological effects of the GABAB receptor has received only limited attention so far. However, it has been demonstrated that targeting GABAB1a sushi domains may impair GABAB receptor function (Tiao et al., 2008). Actually, the most remarkable interaction was recently described between the sushi domains and the shed amyloid-β precursor protein ectodomain APP and its physiological effect was to regulate GABAB1a-mediated regulation of synaptic transmission (Rice et al., 2019).

The fibulin-2 is an extracellular matrix protein that has been shown to interact with the GABAB1a sushi domains with yeast two-hybrids and pull-down assays (Calver et al., 2002; Blein et al., 2004). Fibulin-2 modulates spinal axon growth trajectories during development (Schaeffer et al., 2018). It also mediates pro-neurogenic effects of transforming growth factor-beta1 in adult neural stem cells (Radice et al., 2015). Fibulin2 expression in the central nervous system remains largely unexplored but recent studies indicate it is produced by astrocytes (Radice et al., 2015; Schaeffer et al., 2018). In the present study we identified the colocalization between the GABAB receptor and the fibulin-2 *in situ* in the dorsal spinal cord, and we provide evidence for a direct interaction *in vitro* after the overexpression of tagged GABAB and fibulin-2 proteins.

### Regulation of GABAB Receptor Activation by Fibulin-2

We assessed the effects of fibulin-2 on GABAB receptor activation using FLIM measurement of FRET interactions as previously described (Laffray et al., 2012). FRET provides an accurate measure of possible changes in the distance (<10 nm) and/or orientation between the fluorophores. Hence, it allows to study conformational changes between fluorescently tagged GABAB1a (GABAB1a-GFP) and GABAB2 (GABAB2-DsRed) receptor subunits (Fowler et al., 2007; Laffray et al., 2007). Baclofen-induced increase in FRET efficiency between GABAB1a and GABAB2 was demonstrated by others (Laviv et al., 2010). In contrast, our own studies indicated that the FRET efficiency between GABAB1b and GABAB2 is not, or slightly affected by baclofen (Laffray et al., 2012) (see also Figure 2D of the present study), probably because the absence of sushi domains in the GABAB1b subunit that changes the distance and/or the orientation of the GFP fused at the N-terminal of the B1a/b subunits.

Fibulin-2 limits GABAB activation as demonstrated with FRET analysis and GTP binding assay. High concentration or overexpression of fibulin-2 is likely to increase the number of GABAB receptors interacting with the extracellular matrix protein, therefore impairing the overall modulatory effect of the receptor. In contrast to the action of other GABAB partner proteins, such as 14-3-3 (Laffray et al., 2012), fibulin-2 is unlikely to regulate the dimeric state of the receptor according to the co-immunoprecipitation results. Therefore, we favored the hypothesis that fibulin-2 regulates the receptor conformation. In line with this hypothesis, the higher FRET efficacy upon anti-fibulin-2 siRNA treatment indicated that less constraints apply to the GABAB receptor subunits in the absence of fibulin-2, thus increasing the probability of conformational changes and receptor activation. Importantly, these data also demonstrated that endogenous fibulin-2 exerted tonic inhibition of GABAB activation.

Other examples of interactions with extracellular matrix proteins have been shown to regulate receptor activation. Tenascin R interacts with the B1b subunit and blocks post-synaptic-mediated GABAB inhibition on pyramidal neurons of the hippocampus via the HNK-1 carbohydrate (Saghatelyan et al., 2003). The NMDA glutamate receptor interacts with the matrix protein Reelin that regulates NMDA synaptic retention and surface distribution (Groc et al., 2007). The activity of AMPA receptors and their function in short-term synaptic plasticity also depends on the interaction between receptors and extracellular matrix proteins (Frischknecht et al., 2009).

It may be hypothesized that fibulin-2 acts through two different modes of action, i.e., alteration of ligand affinity or receptor conformation. However, fibulin-2 has different effects on the action of agonists (GABA and baclofen) and competitive antagonists (saclofen and CGP), although they interact with the same binding site. Hence, fibulin-2 is unlikely to simply modify ligand binding to the receptor. This is further confirmed by the competition binding assay (Figure 3C) that indicates there is no changes in the ligand affinity. The extracellular matrix protein may rather alter the receptor conformation, thus changing the GG-protein-mediated effects of agonists.

One can propose that the interaction between fibulin-2 and the Sushi domains themselves influence the efficiency of ligands. Indeed, a regulatory role of the Sushi domains has been shown for glucagon and VIP receptors where the N-terminal extracellular domain of receptor contains highly conserved amino acid residues which are essential for its intrinsic binding activity (Carruthers et al., 1994; Couvineau et al., 1995).

Another possible mechanism relies on fibulin-2 dimerization (Sasaki et al., 1997). As a dimer, fibulin-2 could then bridge two GABAB heterodimers with each other. The formation of such higher order GABAB oligomers (Pin et al., 2009; Comps-Agrar et al., 2011) could account for limitations of structural changes and of GABAB activation.

Alternatively, the interaction between fibulin-2 and the Sushi domains may also interfere with the receptor trafficking and regulate GABAB inhibition as demonstrated for ATF4, another interactor (Corona et al., 2018).

### Consequences of Fibulin-2/GABAB Interaction in Pain Sensitization

Nerve injury leading to pain sensitization has already been shown to induce up-regulation of extracellular matrix proteins in adult rats. More precisely, peripheral lesions increase the expression of fibronectin in the dorsal spinal cord, ipsilateral to the lesion (Nasu-Tada et al., 2006; Tsuda et al., 2008). Such injury-induced overexpression of matrix proteins is involved in pain sensitization by driving an increase of P2X4 receptor expression on microglia (Tsuda et al., 2003, 2008; Ulmann et al., 2008). Interestingly, a recent transcriptomics study indicates that the extracellular matrix pathways are the most largely regulated pathways in animal models of chronic pain, and these data are corroborated by data on human low back pain (Parisien et al., 2019), thus strengthening the hypothesis that the extracellular matrix plays a major role in pain sensitization processes.

The B1a/B2 heterodimer is mainly distributed in the presynaptic compartment (Vigot et al., 2006; Biermann et al., 2010). In superficial laminae of the spinal cord, the GABAB receptor presynaptic inhibition is more effective to suppress mechanical noxious transmission than innocuous transmission, which may account for a part of the mechanism of the analgesic effects of baclofen (Fukuhara et al., 2013). These results suggest that GABAB receptors tonically inhibit glutamate release from primary sensory fibers at a subset of synapses in deep dorsal horn, being more specific of the early phase of synaptic excitation (Salio et al., 2017). In agreement with this presynaptic localization, we showed that fibulin-2 controlled presynaptic inhibition mediated by the GABAB receptor. Therefore, we conclude that fibulin-2 exerts it action through interactions with the presynaptic receptor subtypes, although post-synaptic effect cannot be ruled out. In the spinal dorsal horn, we also showed that fibulin-2 is expressed in superficial laminae, in the vicinity of CGRP-containing nerve endings. The colocalization between these markers indicates that fibulin-2 can exert its regulatory effect on primary nociceptive afferents. Fibulin-2 up-regulation could thus limit the GABAB-mediated suppression of neurotransmitter release by sensory primary afferents in the superficial laminae of the dorsal horn. Fibulin-2 could also impede GABAB presynaptic inhibition on interneurons. In line with this role of the extracellular matrix protein, the knockdown of fibulin-2 enhances the efficacy of baclofen to alleviate mechanical allodynia in neuropathic rats. Our morphological data showed that only a subset of B1a subunit is associated to fibulin-2 at the cell surface. In line with previous reports (Frischknecht et al., 2009), our study suggests that the extracellular matrix protein could trap receptors, and control their activation in specific membrane domains. It may also restrain and filter the exchange of receptors between different subcellular compartments.

Taken together, our data indicate that fibulin-2 overexpression induces structural changes in the GABAB receptor that restrain its activation by the endogenous ligand, GABA, thus allowing a fine tuning of the receptor activation in sub-domains of the plasma membrane.

The regulation by the extracellular matrix may also limit the efficiency of therapeutic strategies aiming to stimulate directly the GABAB receptor. Our data are in line with the actual recommendations for clinical use of baclofen. In fact, baclofen administration is not proposed in EFNS guidelines of pharmacological treatment of neuropathic pain (Attal et al., 2006) and its general effectiveness as an analgesic is limited (Bowery et al., 2002). Administration of positive allosteric modulators may increase the effectiveness of baclofen treatment. However, recent studies point to a limited efficacy of GABAB allosteric modulators such as rac-BHFF in neuropathic mice (Zemoura et al., 2016; Malcangio, 2018). To overcome this limitation, co-application of inhibitors of extracellular matrix proteins, or positive allosteric modulators (Zemoura et al., 2016), together with baclofen, might provide a new mean to enhance baclofen efficiency in clinics, without using high doses that produce unwanted side effects such as sedation.

Together with the recent elucidation of other mechanisms involving reduced GABA release (Moore et al., 2002) or loss of GABAA inhibition (Coull et al., 2005; Knabl et al., 2008) our data support the view that disinhibition dramatically twists the excitability of spinal neurons and leads to pain sensitization (Woolf and Salter, 2000). Targeting GABAB associated proteins, and especially extracellular matrix proteins, may be of therapeutic interest by enhancing the action of classical pain killers.

## Data Availability Statement

The raw data supporting the conclusions of this article will be made available by the authors, without undue reservation.

## Ethics Statement

The animal study was reviewed and approved by Comité local d’éthique, Université de Bordeaux.

## Author Contributions

M-AP, ML, RR-P, and FN conceived the study and planned the experiments. M-AP performed the FRET and immunohistochemistry experiments, and the pain behavior studies. GB-G performed the binding and the GTPγS assays. YL and FF performed the patch-clamp recordings. AF performed the qRT-PCR experiments. RB-B performed the co-immunoprecipitation experiments and the pain behavior studies. M-AP, ML, RR-P, YL, and FN wrote the manuscript. All authors contributed to the article and approved the submitted version.

## Conflict of Interest

The authors declare that the research was conducted in the absence of any commercial or financial relationships that could be construed as a potential conflict of interest.

## Footnotes

1 http://www.synaptosoft.com/
